# Genome sequence of the potato pathogenic fungus *Alternaria solani* HWC-168 reveals clues for its conidiation and virulence

**DOI:** 10.1186/s12866-018-1324-3

**Published:** 2018-11-06

**Authors:** Dai Zhang, Jia-Yu He, Parham Haddadi, Jie-Hua Zhu, Zhi-Hui Yang, Lisong Ma

**Affiliations:** 1 Center of Plant Disease and Plant Pests of Hebei Province, College of Plant Protection, Hebei Agricultural University, Baoding, 071001 China; 2 Saskatoon Research and Development Centre, Agriculture and Agri-Food Canada, Saskatoon, SK S7N0X2 Canada

**Keywords:** *Alternaria solani*, Genome sequence, Secretome, Conidiation, Virulence

## Abstract

**Background:**

*Alternaria solani* is a known air-born deuteromycete fungus with a polycyclic life cycle and is the causal agent of early blight that causes significant yield losses of potato worldwide. However, the molecular mechanisms underlying the conidiation and pathogenicity remain largely unknown.

**Results:**

We produced a high-quality genome assembly of *A. solani* HWC-168 that was isolated from a major potato-producing region of Northern China, which facilitated a comprehensive gene annotation, the accurate prediction of genes encoding secreted proteins and identification of conidiation-related genes. The assembled genome of *A. solani* HWC-168 has a genome size 32.8 Mb and encodes 10,358 predicted genes that are highly similar with related *Alternaria* species including *Alternaria arborescens* and *Alternaria brassicicola*. We identified conidiation-related genes in the genome of *A. solani* HWC-168 by searching for sporulation-related homologues identified from *Aspergillus nidulans*. A total of 975 secreted protein-encoding genes, which might act as virulence factors, were identified in the genome of *A. solani* HWC-168. The predicted secretome of *A. solani* HWC-168 possesses 261 carbohydrate-active enzymes **(**CAZy**)**, 119 proteins containing RxLx[EDQ] motif and 27 secreted proteins unique to *A. solani*.

**Conclusions:**

Our findings will facilitate the identification of conidiation- and virulence-related genes in the genome of *A. solani.* This will permit new insights into understanding the molecular mechanisms underlying the *A. solani*-potato pathosystem and will add value to the global fungal genome database.

**Electronic supplementary material:**

The online version of this article (10.1186/s12866-018-1324-3) contains supplementary material, which is available to authorized users.

## Background

*Alternaria,* a genus of ascomycete fungi, causes various disease symptoms, including root and stem rot, blight, and wilt on most economically important plants [1]. *Alternaria solani* is known as the causal agent of early blight of potato and tomato. Early blight of potato is a major foliar disease that is considered one of the most destructive diseases of potato worldwide, resulting in severe yield losses in many potato growing regions [1].

Understanding the factors influencing spore formation and identification of a wide range of secondary metabolites produced by *A. solani* have been the subject of extensive studies in the past many years. For example, Brian et al. first reported that Alternaric acid is a biologically active product of the fungus *Alternaria solani* [2, 3]. In addition, *A. solani* is capable of producing extracellular polysaccharides, carbohydrases, proteases, the new zinniol-related phytotoxins [4, 5], and other secondary metabolites during infection [6, 7]. It has been documented that sporulation of *A. solani* depends upon many factors such as mycelial wounding, temperature, visible light, water treatment, ozone and ultraviolet [8–11]. Growth characteristics, genetic and pathogenic variations of *A. solani* have been studied as well [12–17]. Based on these successful and progressive studies on *A. solani*, the interaction between *A. solani* and its host represents an excellent system that will enable researchers to study the pathogenic mechanisms between *Alternaria* species and their hosts.

Conidiation (asexual sporulation) in filamentous ascomycetous fungi is a complex process involving the formation of conidia on conidiophores [18]. Many studies have been conducted to investigate the sporulation process, resulting in the identification of various environmental factors influencing sporulation, such as light, salt and nutrients and endogenous biological rhythms, but the light is regarded as one of the key environmental factors for regulating sporulation [19]. The molecular basis underlying the conidiation of *Aspergillus nidulans* and *Neurospora crassa* has been well studied, leading to the identification of a set of genetic regulators controlling the asexual sporulation in *A. nidulans* [20–22]. Activation of the transcription factor *brlA* gene by light has been demonstrated as an essential step of conidiation in *A. nidulans* [22, 23]. *abaA* gene is activated by BrlA and loss of *abaA* results in the formation of supernumerary tiers of cells with abacus-like structures [18, 24]. *wetA* gene induced by AbaA during the late stage of conidiation activates a set of genes responsible for the synthesis of cell wall layers and spore specific functions [25, 26]. These three sequentially expressed genes including *brlA*, *abaA* and *wetA* comprise a central regulatory pathway that controls the sporulation in *A. nidulans* [18, 24]. In addition to these three genes, six upstream developmental activators (*fluG*, *flbA*, *flbB*, *flbC*, *flbD* and *flbE*) have been identified by genetic studies on recessive mutations to cause defective conidiation [27]. StuA regulates transcription of the *brlA* gene and plays a key role in the structure and cell morphogenesis during the sexual and asexual phases of reproduction in *A. nidulans* [28, 29]. Previous studies have reported that nutrition, light spectrum and temperature are major factors that influence the sporulation of *A. solani* in vitro; however, the production of conidiospores is limited and variable among distinct isolates of *A. solani* and there is no a common practical protocol developed for the species. Therefore, understanding the molecular mechanism underlying the conidiation of *A. solani* is urgently required.

Advances in next-generation sequencing (NGS) technologies are transforming biology research. The large-scale studies of fungal genome sequence have facilitated the discovery of molecular mechanisms underlying the virulence in plant fungal pathogens. Recently, several genome sequences of *A. solani* isolates have been reported including BMP0185, CBS109157 and altNL03003 [30–32]. Interestingly, the genome of *A. solani* altNL03003 isolated from a Dutch potato field has been sequenced using the long-read Pacific Biosciences (PacBio) sequencing technology. This has provided a gapless genome assembly and produced a genome size of 32.8 Mb [32]. The available *Alternaria* genome sequence database provides a useful resource to browse and visualize whole genome alignments, genome annotations, and identify homologous genes within the important saprophytic and plant/human pathogenic fungal genus [18, 33–36]. However, a detailed genome annotation and prediction of genes encoding secreted proteins remain unknown for *A. solani*, especially in the genome of *A. solani* isolate from China. Here, we present an accurate genome annotation and provide a prediction of conidiation and effector candidate genes from *A. solani* HWC-168*,* which holds the potential to advance our understanding of pathogenic mechanisms of *A. solani*.

## Results

### Genome sequencing and assembly

To gain a better understanding of *A. solani* genome, we generated a high-quality genome sequence of *A. solani* HWC-168 using an Illumina HiSeq 2000 sequencing platform. The high quality of genomic DNA isolated from the mycelium of *A. solani* HWC-168 was used to prepare libraries. Two independent DNA libraries were constructed: one with insert size 500 bp and second one with 5 kb insert size. Total 21.9 Gb and 33.7 Gb of high quality reads were generated from 500 bp library and 5 kb library, respectively. The genome coverage was 200-fold in the library containing insert size 500 bp and 308-fold in the library with 5 kb insert size. The reads generated from both libraries were assembled into 209 contigs and 61 scaffolds, among which the size of the longest scaffold was 5,423,972 bp and scaffold N50 having the size of 2,613,338 bp. The assembled genome size achieved was 32,838,780 bp, which agrees favorably with the reported genome size of *A. solani* of 32.6 to 32.9 Mb [31, 32]. The GC content of the genome was 51.20% of the total bases (Table 1).

**Table 1:** Summary of genome assembly and annotation features of *A. solani*, *A. arborescens* and *A.brassicicola*

	*A. arborescens* EGS39–128	*A. brassicicola* ATCC 96836	*A. solani* HWC-168	*A. Solani* altNL03003
Genome				
Isolate	EGS 39–128	ATCC 96836	HWC-168	altNL03003
Coverage (fold)	90	6.4	508	> 150
Genome size	33,889,384 bp	29,536,471 bp	32,838,780 bp	32,779,142 bp
No. of contigs	1332	4039	209	10
Largest contig length	1,056,452 bp	87,976 bp	1,075,562 bp	7,235,174 bp
N50 contig length	310,869	18,835	564,368	
G + C content (%)	50.9	50.7	51.2	51.2
Genes				
Number of genes	11,042	10,514	10,358	
Gene density(genes/Mb)	325	356	323	
Number of specific genes	1689	1883	1632	

### Repeat content in *A. solani* HC-168

To characterize the assembled genome, the repetitive elements were identified using the CENSOR prediction. In total, 24,896 repeat elements including DNA transposon, endogenous retrovirus, LTR retrotransposon, non-LTR retrotransposon, pseudogene, satellite and integrated virus were identified in the genome (Table 2). Our analysis revealed that the repeat content accounted for 6.95% of the gnome in length, which differs with that of *A. solani* CBS109157 [31]. The distribution of LTR retrotransposon was heavily dominant (3.3% of the entire genome) but DNA transposon (2.43%), Non-LTR retrotransposon (0.91%) and endogenous retrovirus (0.21%) were also highly represented. Based on the superfamily types, most common types of repetitive elements were represented in the *A. solani* genome with the dominant family being Gypsy (2.7% of the genome) and the most abundant family being Copia (0.67%) and EnSpm/CACTA (0.60%) and Mariner/Tc1 (0.45%).

**Table 2:** Summary of repetitive elements present in the genome of *A. solani* HWC-168

Repeat element	Superfamilies	Number of elements	Length occupied	% of assembled genome
DNA transposon		7633	798,198	2.43
	EnSpm/CACTA	1640	198,077	0.60
	Hobo-Activator (hAT)	1139	100,772	0.31
	Mariner/Tc1	901	148,218	0.45
	MuDR	754	60,332	0.18
	Harbinger	687	52,748	0.16
	Polinton	476	59,121	0.18
	Helitron	374	27,301	0.08
	Kolobok	233	33,732	0.10
	Sola	210	20,091	0.06
	Crypton	146	10,037	0.03
	piggyBac	132	8575	0.03
	Dada	116	9219	0.03
Endogenous Retrovirus		1069	69,620	0.21
	ERV1	414	27,788	0.08
	ERV2	360	23,610	0.07
	ERV3	102	6650	0.02
	ERV4	73	4385	0.01
LTR Retrotransposon		11,814	1,074,479	3.30
	Gypsy	6875	745,838	2.27
	Copia	3378	220,000	0.67
	BEL	989	58,924	0.18
	DIRS	339	21,781	0.07
Non-LTR Retrotransposon		4109	297,677	0.91
	L1	1049	66,695	0.20
	CR1	538	44,451	0.14
	R1	318	24,513	0.07
	Jockey	287	21,278	0.06
	RTE	232	15,218	0.05
	Tx1	211	14,176	0.04
	I	175	11,361	0.03
	Tad1	161	11,574	0.04
	L2	121	8125	0.02
	RTEX	106	8159	0.02
Pseudogene		94	16,566	0.05
	rRNA	57	13,659	0.04
	tRNA	37	2907	0.01
Satellite		145	14,774	0.04
	MSAT	40	6529	0.02
	SAT	63	4970	0.01
Integrated Virus		32	1760	0.005
	Caulimoviridae	29	1516	0.004
	DNA Virus	3	244	0.001
TOTAL		24,896	2,273,074	6.95

### Comparison of genome assembly features within *Alternaria* species

**Fig. 1:**
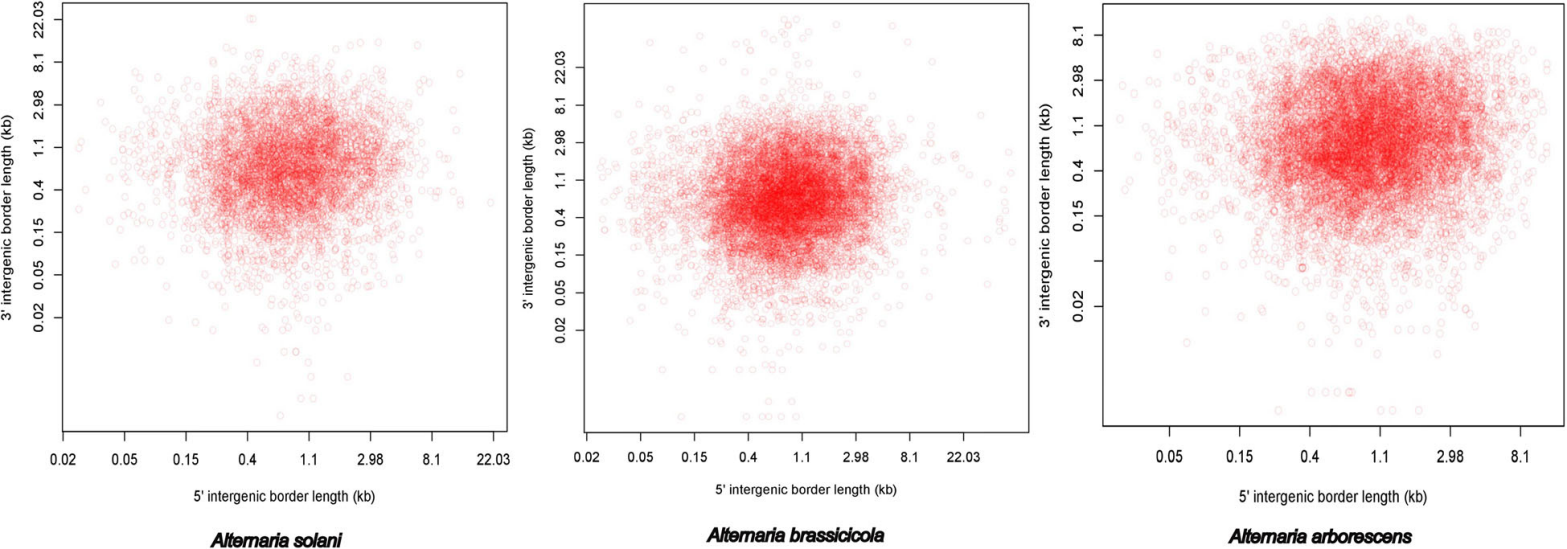
Distribution of intergenic distances of all predicted genes present in the genome of *A. solani* HWC-168 compared with *A. arborescens* EGS 39–128 and *A. brassicicola* ATCC 96836. Scatterplot representing 5′ and 3′ intergenic distances for all genes present in the genome. Red circles indicate the predicted genes

The genome size of *A. solani* HWC-168 (32.8 Mb) was small compared to *A. arborescens* (33.9 Mb) but larger than *A. brassicicola* (29.5 Mb) [30]. It has approximately the same size as reported for *A. solani* altNL03003 (32.8 Mb) [32]. The average gene density in *A. solani* HWC-168 genome was 323 genes per Mb, which remains lower than that in *A. brassicicola* (356 genes per Mb) and *A. arborescens* (325 genes per Mb). Next, we compared the whole genome assembly features of *A. solani* HWC-168 with those of sequenced *A. arborescens* EGS 39–128, *A. brassicicola* ATCC 96836 and *A. solani* altNL03003 genomes (Table 1)*.* Compared to *A. arborescens* and *A. brassicicola*, our genome assembly was superior because it featured the greatest genome coverage, the minimum number of contigs and largest N50 contig length (Table 1). However, compared to *A. solani* altNL03003, a large number of contigs was observed in our assembly (Table 1). In addition, we compared the gene distribution in the three annotated *A. brassicicola*, *A. arborescens* and *A. solani HWC-168* genomes by calculating the intergenic distance between adjacent genes. Figure 1 showed that the distributions of intergenic distances in *A. solani* HWC-168 genome were similar to those in the genome of *A. brassicicola*. However, the distributions of intergenic distances in *A. arborescens* genome were less variable and genes in *A. arborescens* genome were more closely spaced than the analyzed *A. solani* and *A. brassicicola* genomes (Fig. 1).

### Gene prediction and functional annotation

To predict complete genes in *A. solani* HWC-168 genome, we used the Augustus version 2.5.5 [53]. The analysis resulted in 10,358 complete genes in the genome of *A. solani* HWC-168. PanOCT analysis was employed to examine the orthologous gene clusters among predicted genes of *A. solani* HWC-168, *A. arborescens* EGS 39–128 and *A. brassicicola* ATCC 96836. The total number of predicted genes, core genes, clusters of orthologous groups (COGs) and shared COGs were summarized in the Venn diagram for ortholog clusters in these three genomes (Additional file 1). The three genomes shared a core set of 3460 COGs and 6879 core genes. In addition, there were 8304 genes shared between *A. solani* HWC-168 and *A. arborescens* EGS 39–128*,* which was higher than that between *A. solani* HWC-168 and *A. brassicicola* ATCC 96836 (7301), and also higher than that between *A. arborescens* EGS 39–128 and *A. brassicicola* ATCC 96836 (7204). Taken together, these observations strongly indicate that significant gene variations including gene numbers and COGs exist in these closely related *Alternaria* strains, suggesting that these three *Alternaria* strains might have diverged in the genome evolution.

**Fig. 2:**
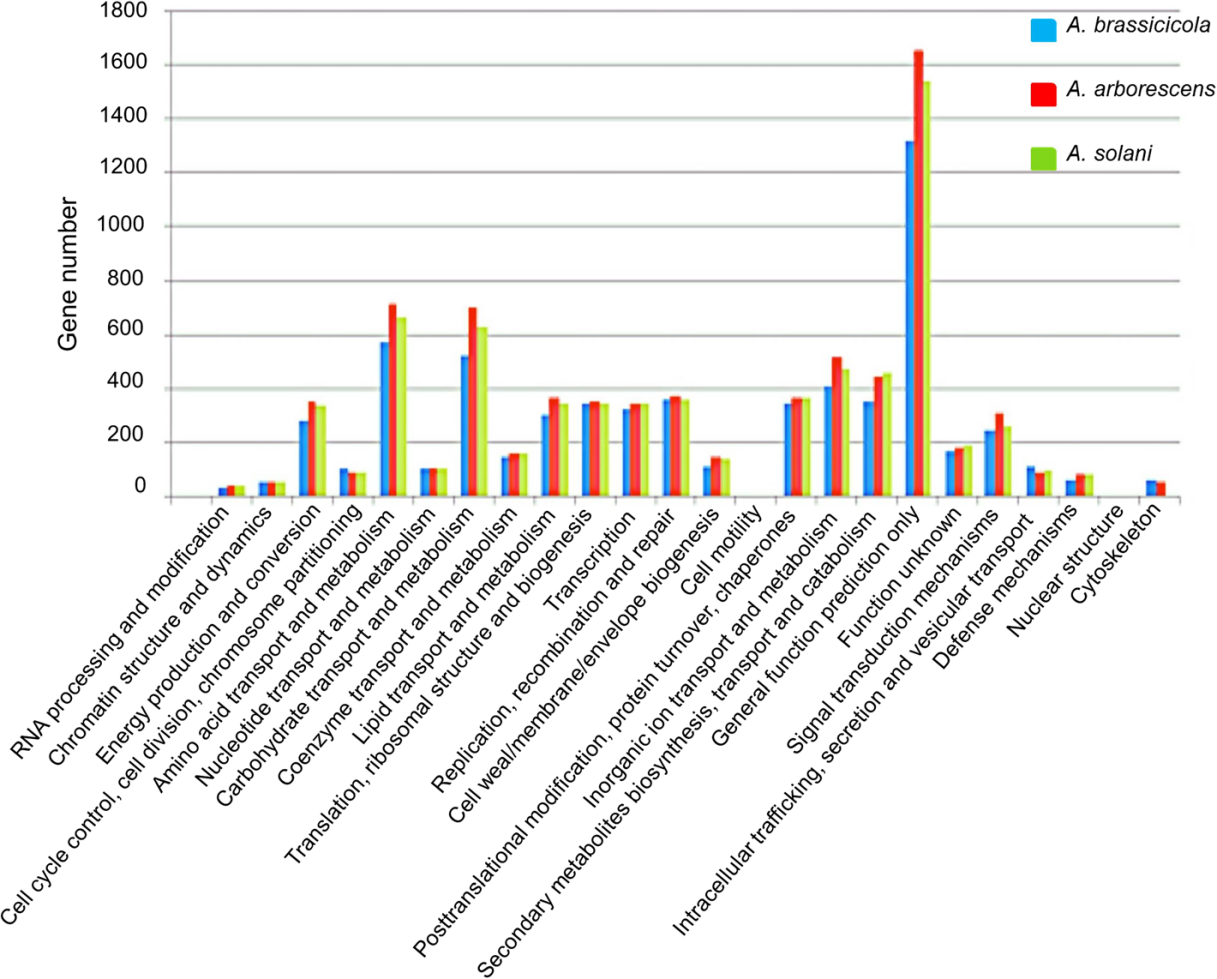
Gene Ontology (GO) classification of genes predicted from the genome of *A. solani* HWC-168, *A. arborescens* EGS 39–128 and *A. brassicicola* ATCC 96836. Predicted genes are assigned to 24 categories in the GO classification. The x-axis legend shows a description of the 24 functional categories and the y-axis indicates the number of genes in a specific function cluster. Among the 24 categories, the cluster of ‘general function prediction’ has the highest number of genes, followed by amino acid transport and metabolism and carbohydrate transport and metabolism

**Fig. 3:**
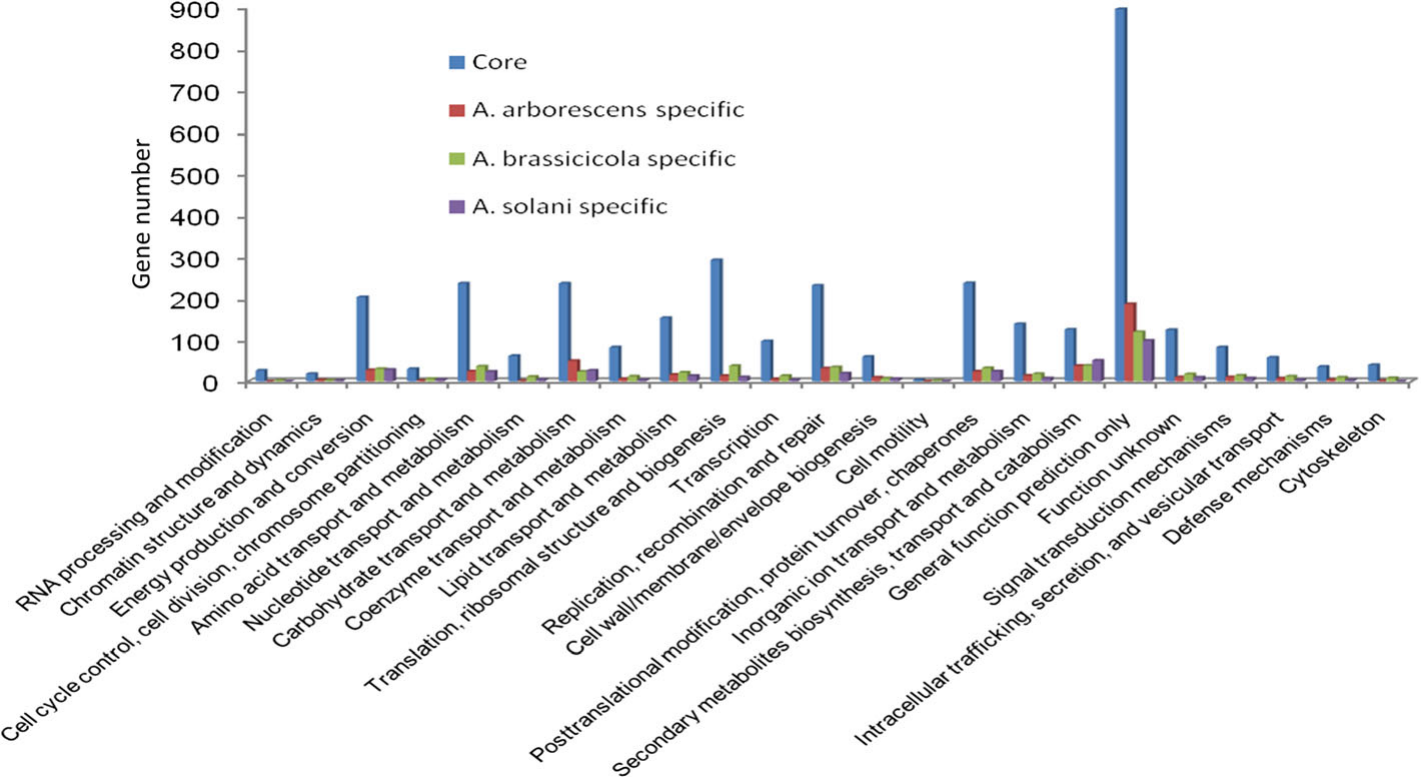
Gene Ontology (GO) classification of core and species-specific genes identified from comparison of *A. solani* HWC-168, *A. arborescens* EGS 39–128 and *A. brassicicola* ATCC 96836 genomes. Predicted core and species-specific genes are assigned to 24 categories in the GO classification. The x-axis legend shows a description of the 24 functional categories and the y-axis indicates the number of genes in a specific function cluster. Among the 24 categories, the cluster of ‘general function prediction’ contains the highest number of core and species-specific genes, followed by translation, ribosomal structure and biogenesis

To annotate the predicted genes and assign Gene Ontology (GO) functions to them, predicted proteins from *A. arborescens* EGS39–128, *A. brassicicola* ATCC 96836 and *A. solani* HWC-168 were searched for homology to entries in the NCBI Ref Seq protein database, GO and InterPro databases using Blast2Go-PRO, respectively. As shown in Fig. 2, annotated genes contributing to the general function, amino acid transport and metabolism and carbohydrate transport and metabolism were predominant within the comparison of GO terms of three *Alternaria* genomes. However, further comparison of GO terms between these three *Alternaria* isolates revealed that the number of genes from each GO category was similar between *A. solani* HWC-168, *A. arborescens* EGS 39–128 and *A. brassicicola* ATCC 96836. In addition, we analyzed the GO functions of core genes and species-specific genes between *A. solani* HWC-168, *A. arborescens* EGS 39–128 and *A. brassicicola* ATCC 96836. A functional GO analysis determined that core genes and species-specific genes involved in general function dominated and that the second most abundant genes were related to translation, ribosomal structure and biogenesis (Fig. 3).

### Secretome of *A. solani* HWC-168

By using SignalPv4.0, TMHMM-2.0, TargetPv1.01, and big-PI Predictor, we searched the genome of *A. solani* HWC-168 for secreted protein-encoding genes, which might act as effector candidate genes. Nine hundred seventy five secreted protein-encoding genes were identified, which accounted for 9.4% of the total predicted genes.

#### Cell wall degrading enzymes

The majority of the secreted proteins were identified as cell wall degrading enzymes (CWDEs) involved in plant cell degradation. In addition, other enzymes that participate in various cellular metabolisms and non-enzyme proteins that maintain cellular energy and transport were also identified among these secreted proteins. Interestingly, we found that some secreted proteins identified from the secretome of *A. solani* HWC-168 were assigned to the same functional annotation but had differing functional classification (Additional file 2), suggesting that these secreted proteins may play important roles in various cellular activities*.*

#### Carbohydrate-active enzymes and proteins with other predicted functions

**Fig. 4:**
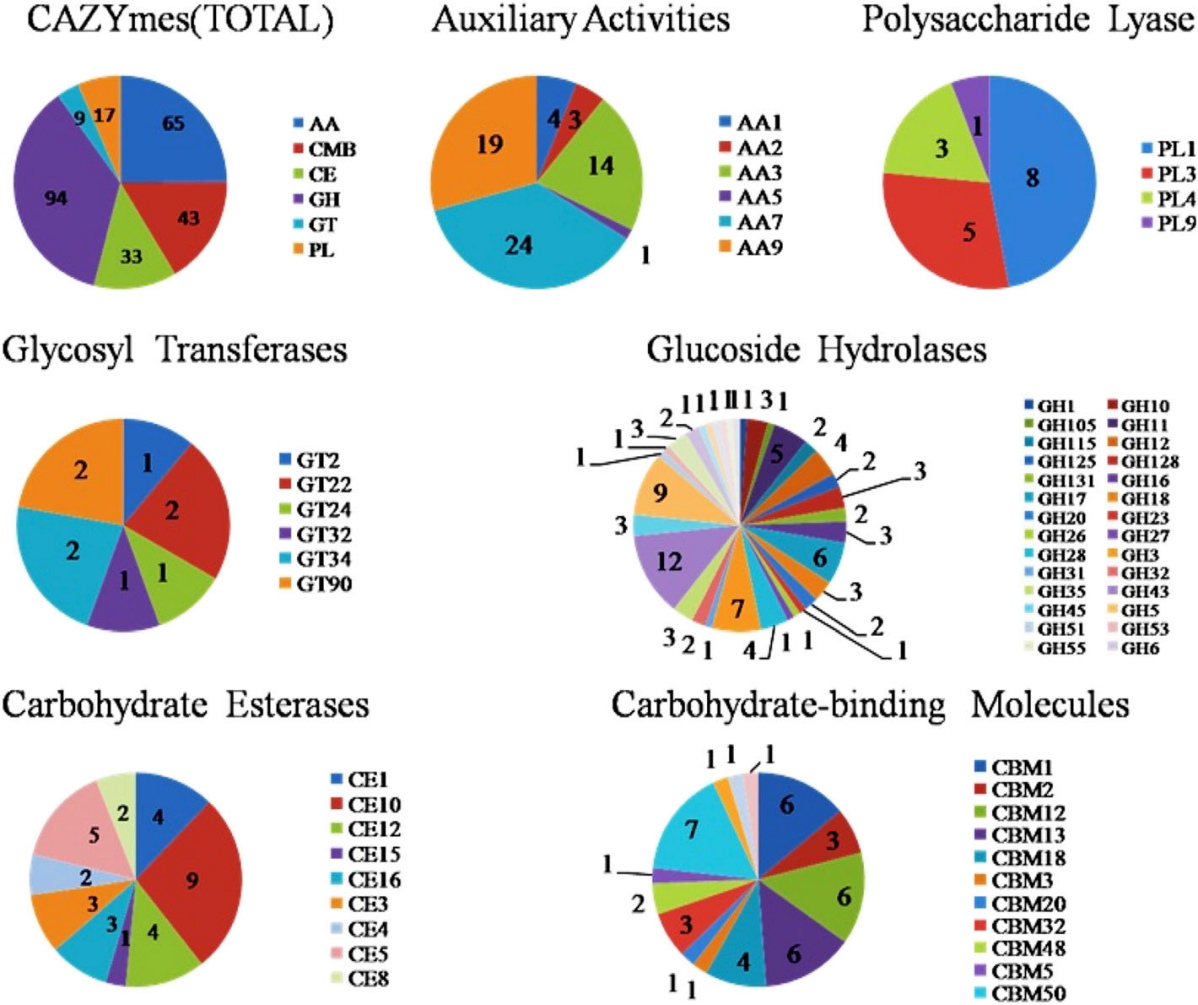
Graphical representation of predicted carbohydrate-active enzymes encoding genes in the genome of *A. solani* HWC-168. Total 261 predicted CAZymes are identified and they are divided into six sub-groups including 65 auxiliary activity (AA), 17 polysaccharide lyase (PL), 9 glycosyl transferase (GT), 94 glucoside hydrolase (GH), 33 carbohydrate esterases (CE) and 17 carbohydrate-binding molecules (CBM). Glucoside hydrolase is predominant in all predicted CAZymes

The *A. solani* HWC-168 secretome possessed 261 secreted carbohydrate-active enzymes (CAZymes) with predicted activities (Fig. 4). One protease and one SnodProt elicitor belonging to the cerato-platanin protein (CPP) family were identified within the secretome of *A. solani* HWC-168. Surprisingly, a secreted protein exhibiting sequence homology to a superoxide dismutase was identified in the secretome of *A. solani* HWC-168. It has been reported that the superoxide dismutase is involved in inhibiting oxidative damage of pathogens and plant resistance [37, 38]. Furthermore, three trihydrophobins that are commonly found in the surface of aerial hyphae or fruiting body in fungi were predicted to be secreted [14, 15]. The presence of trihydrophobins in the secretome of *A. solani* HWC-168 suggests their potential roles in fungal development, morphological differentiation and pathogenicity.

#### RxLx[EDQ] effector candidates

**Fig. 5:**
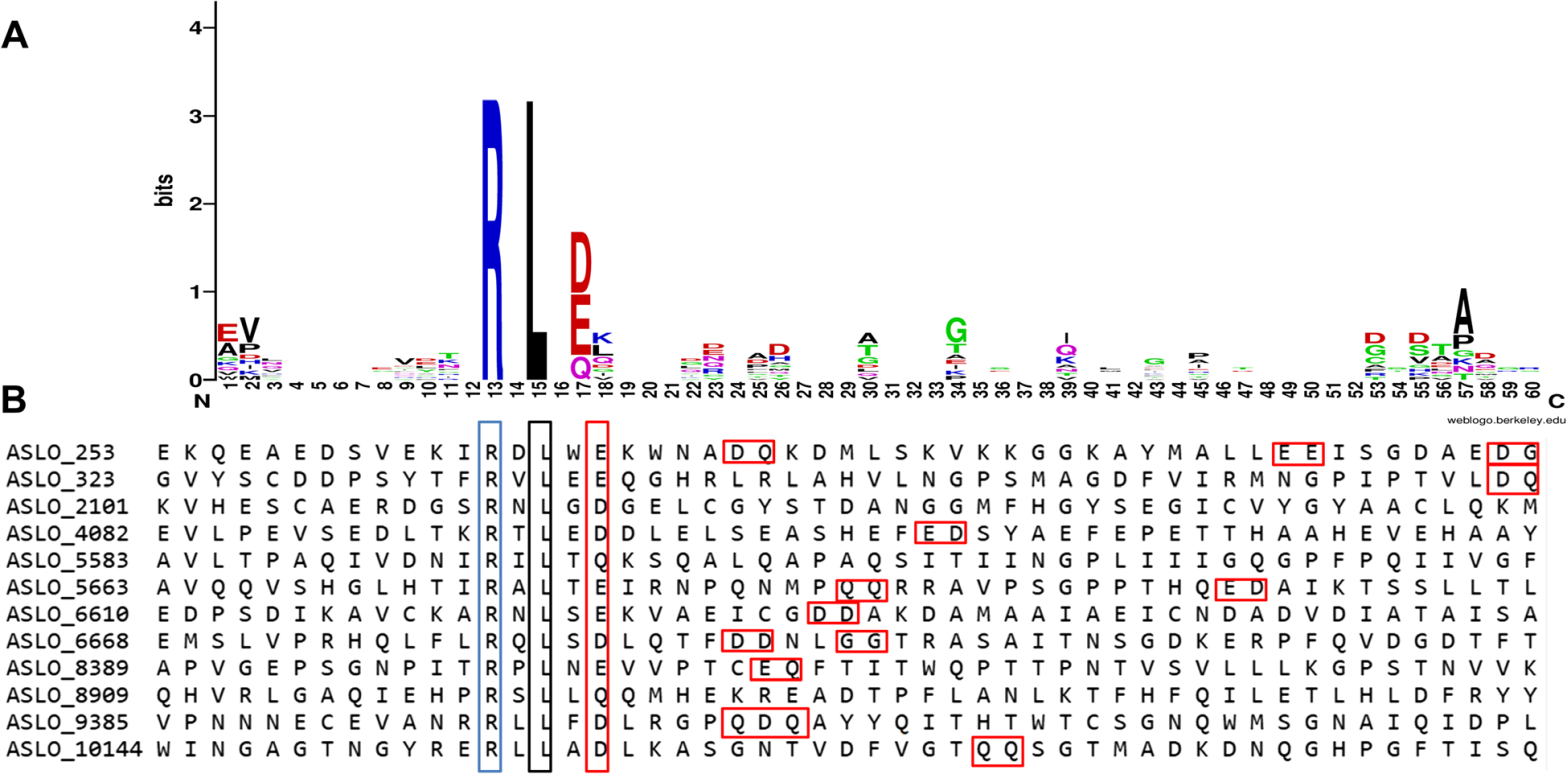
Schematic representation of amino acid sequences alignment of 12 RxLx-motif containing effector candidates. **a**. Sequence logo derived from 12 predicted secreted effector candidates carrying the RxLx[EDQ] motif located within the region of 120 amino acids downstream of the N-terminal signal peptide. **b**. the conserved amino acids in the RxLx[EDQ] motif are highlighted and the downstream EDQ amino acids are marked with a red rectangle

The RxLx[EDQ] motif functions as a host-targeting signal to deliver virulence proteins of *Plasmodia falciparum* into host cells [39]. The secretome of *A. solani* HWC-168 contained 119 secreted proteins possessing the RxLx[EDQ] motif (where x represents any amino acid). One of important criteria for effector prediction appears to be protein size less than 300 amino acids. Based on this criterion, 12 effector candidate proteins carrying RxLx[EDQ] motif within 120 amino acids downstream of N-terminal signal peptide were identified (Table 3). WEBLOGO analysis revealed that amino acids Arginine (R) in position 1 and Leucine (L) in positon 3 and glutamic acid (E)/ aspartic acid (D)/glutamine(Q) in the 5 position were highly conserved in the RxLx [EDQ] motif. By contrast, bilateral amino acid sequences surrounding the RxLx[EDQ] motif were not conserved and tended to be highly variable (Fig. 5a). In addition, we found that the continuous aspartic acid (D), glutamic acid (E) and glutamine (Q) residues were present in the downstream of the RxLx[EDQ] motif but with variable locations (Fig. 5b). Due to the important roles of RxLR effectors in the pathogenicity of *Phytophthora infestans*, functional analysis of the RxLx[EDQ] motif-containing proteins in the secretome of *A. solani* HWC-168 will be the focus for future research.

**Table 3:** List of predicted effector candidates carrying the RxLx[EDQ] motif

Name	Size	Start of sequence
ASLO_253	148	MKFTLAIVALASVATTALANREWTYNDSHRAAVTAILKQITAKHAHLCKR
ASLO_323	134	MKLAIAALFASITAAAPTATPDVHGDPFETVTISNFVYVGVNGYPQIDFH
ASLO_2101	114	MQIMNLAVLAATLATVGAWTLDDYGKWVANNAWRDNLNGVHKVHESCAER
ASLO_4082	266	MKFLAIIVAAQLATALPVAKEACSSTDITCSATKANPQIFDVASQQLDNS
ASLO_5583	205	MVSFRNLFTAAMALSVPVAAVLTPAQIVDNIRILTQKSQALQAPAQSITI
ASLO_5663	199	MASSIPDHWLWLGLGVFTFIAVQQVSHGLHTIRALTEIRNPQNMPQQRRA
ASLO_6610	169	MLGRTVFAATFFALAQFAMASPPSCLLGAVNQYEDPSDIKAVCKARNLSE
ASLO_6668	183	MFLPTALLALVHLALPALSHASPQPALVSSDWEMSLVPRHQLFLRQLSDL
ASLO_8389	252	MYSKTAIVTFFAGFAAAQIHAPVGEPSGNPITRPLNEVVPTCEQFTITWQ
ASLO_8909	270	MAKLIDLSTEVLFLIVAYFTSGDASDVQALLHLCRTSRMLVAVAQPALYT
ASLO_9385	163	MHFSVIFSAVFAATAMAAPASLDSRGDEDCVPDSYTISDYTLITSPTSGS
ASLO_10144	244	MLSNLMNRFALPLAILAFFLSFANGLPHDALIARRTTNLRILPLGDSITW

#### Unique secreted proteins

We observed that 27 predicted genes encoding secreted proteins were completely absent in the genome of *A. arborescens* and *A. brassicicola* (Additional file 3)*.* As a consequence, the function of these secreted proteins remains unknown due to the lack of homology with known proteins. Interestingly, from 27 species-specific genes we identified 3 pairs of neighbor genes that reside on three different scaffolds: scaffold 18, scaffold 21 and scaffold 8, respectively (Additional file 4). These findings suggest that the presence of these species-specific secreted protein-encoding genes in the genome of *A. solani* HWC-168 may have originated by two possibilities: either the genome of *A. solani* HWC-168 possesses a large genomic fragment that is missing in other *Alternaria* genomes or the genomic databases of *A. arborescens* and *A. brassicicola* are incomplete because only their draft genomes are reported, which has resulted in the absence of these secreted proteins. Functional analysis of these species-specific secreted proteins in *A. solani* HWC-168 is under way in our laboratory.

### Prediction of conidiation-related genes

**Fig. 6:**
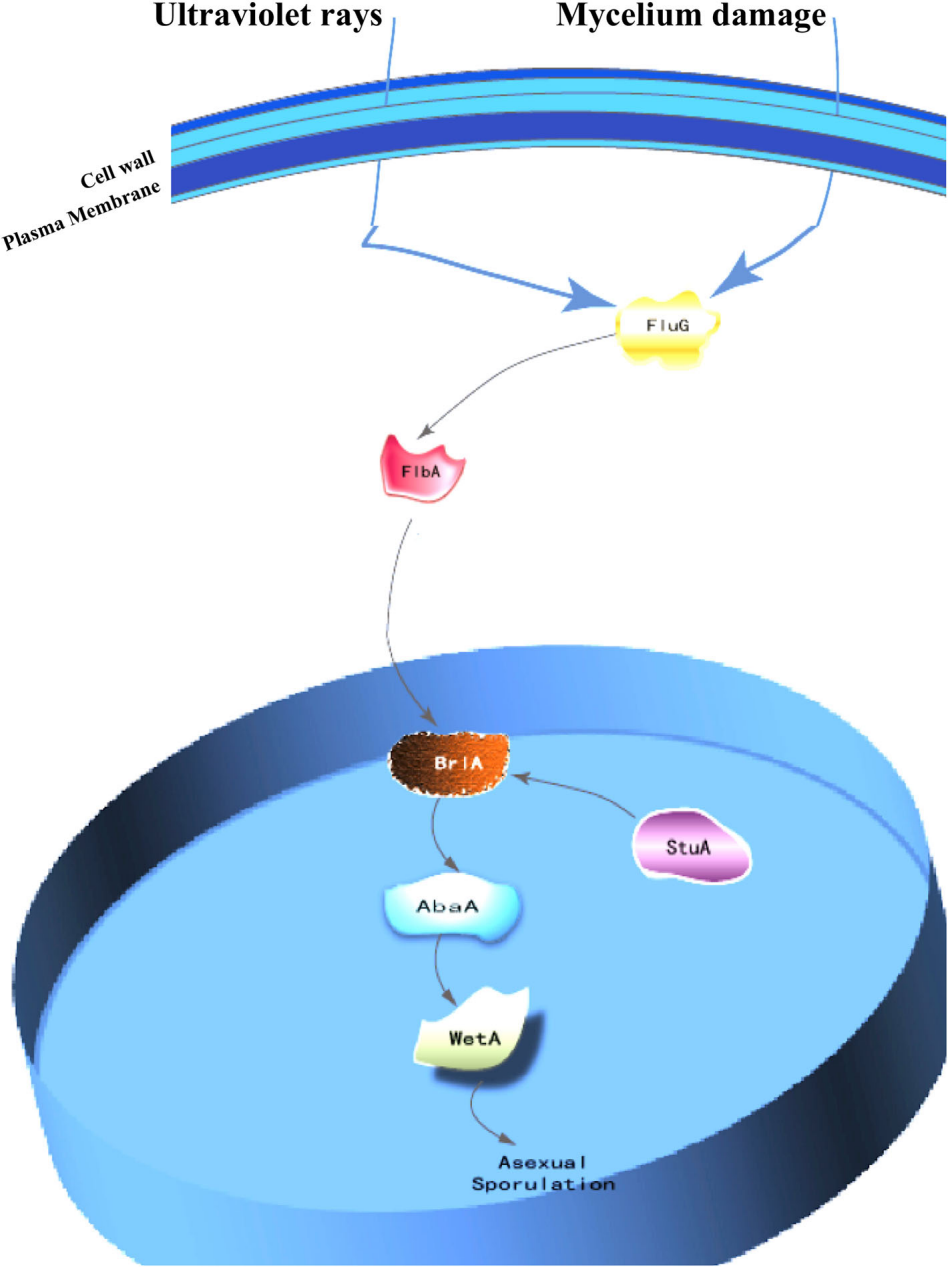
Schematic representation of a proposed model illustrating the regulatory pathway of asexual sporulation in *A. solani*

Our earlier studies revealed that it was difficult to induce conidiation in our *A. solani* isolates under artificial culturing condition. However, *A. solani* HWC-168 is capable of yielding copious conidiospores when its mycelia are radiated by ultraviolet (UV light) (Additional file 5). To identify genes involved in conidiation, we retrieved the central regulatory genes participating in the conidiation from the genome of *Aspergillus nidulans* and blasted the *brlA* sequence and other sporulation related genes, such as *abaA, wetA, stuA, fluG, flbA, flbC, flbD, flbE, medA a*nd *fadA,* to the predicted protein database of *A. solani.* Our results showed that homologous genes including *fluG*, *flbA*, *flbC*, *flbE*, *brlA*, *stuA*, *abaA, wetA*, *medA* and *fadA* existed in the genome of *A. solani-*HWC168. Based on the proposed conidiation model in *Aspergillus nidulans* and the identified conidiation-related genes in *A. solani* HWC-168, we propose a potential conidiation pathway in *A. solani* HWC-168 (Fig. 6).

## Discussion

Pathogenic *A. solani* strains are increasingly posing a critical threat to world food security. Sequencing the whole genome of *A. solani* is a key step to facilitate the study of the molecular mechanisms underlying the interaction between potato and *A. solani*. Here, we presented the completed genome sequence and annotation of *A. solani* HWC-168 generated by advanced next-generation Illumina sequencing technology. The quality of the genome sequence was guaranteed by using two individual sequencing libraries. The genome sequence data of *A. solani* HWC-168 has the potential to facilitate a future study on the molecular basis of *A. solani* virulence.

Genomes of three *A. solani* isolates have been sequenced in the past [30–32]. The assembled genome size of *A. solani* ranges from 32.6 to 32.9 Mb. Our sequenced *A. solani* HWC-168 produced a genome size of 32.8 Mb, which compares favorably with the reported genome size of *A. solani* altNL03003 (Table 1). The genome of *A. solani* altNL03003 had been sequenced using the long-read Pacific Biosciences (PacBio) sequencing technology, which provided a gapless genome assembly. Although we sequenced the genome of *A. solani* HWC-168 using the second-generation sequencing technology, the same genome size was produced with *A. solani* altNL03003 but the assembled contig number of *A. solani* HWC-168 being higher than that of *A. solani* altNL03003. This suggests a high-quality genome assembly of altNL03003 has been obtained. However, we were not able to compare our predicted genes with those of *A. solani* altNL03003 because of the lack of annotation data of *A. solani* altNL03003. By searching our predicted RxLx[EDQ] effector candidates and conidiation-related genes in the genome of *A. solani* altNL03003, we found that all of them were present in the genome of *A. solani* altNL03003 (Additional file 3 and unpublished data). This observation strongly suggests that the genome annotation of *A. solani* HWC-168 is accurate. It has been reported that the repeat content in the genome of *A. solani* CBS109157 is relatively low with only 1.5% [31]. Surprisingly, we found that the percentage of repeat content of *A. solani* HWC-168 was relatively high with 6.95% although both of them have similar genome size. This apparent discrepancy requires future study to identify repeat contents from the gapless assembled genome of *A. solani* altNL03003.

Asexual sporulation is a common reproduction strategy in filamentous fungi. Although they vary in morphology and function, conidia in higher fungi are developed from specialized sporogenous cells or asexual propagules. The conidiation-related processes are complicated, including the space-time regulation of conidiation-related genes, cell specialization and cell signal transduction, etc. The genetic mechanism of conidiation in *Aspergillus nidulans* has been well studied [40, 41]. However, the molecular basis underlying conidiation in *A. solani* remains unclear. Elucidation of genes regulating the conidiation process is essential to our understanding of asexual reproduction in *A. solani* species. Here, we first presented the evidence that the central regulatory factors of conidiation identified from *Aspergillus. nidulans* including *abaA*, *wetA*, *StuA*, *FluG* and *FlbA* genes are present in the genome of *A. solani* HWC-168. This suggests a similar molecular mechanism of sporulation is employed by *A. solani;* however, these homologous conidiation-related genes in *A. solani* are putative and functional confirmation is required. We are examining the expression profiles of predicted conidiation-related genes (unpublished data). We are confident that future studies designed to elucidate the molecular basis of conidiation will provide the impetus to develop novel strategies to prevent sporulation in order to control disease development caused by *A. solani* on potato.

The secreted fungal enzymes in pathogenic fungi play important roles in pathogenicity. Here, we found that the secreted enzymes in *A. solani* HWC-168 contain a large number of cellulases and pectinases. Previous studies showed that cellulases and pectinases in *Alternaria* species play key roles in degrading the plant cell wall during infection [42–44]. Thus, we proposed that cellulases and pectinases in *A. solani* play important roles in infecting the host and causing degradation of host cell wall. In our work, a total of 975 predicted secreted proteins were identified in the genome of *A. solani* HWC-168, comprising 261 CAZymes, 119 RxLx[EDQ] motif containing secreted proteins and 27 species-specific secreted proteins. It remains unknown how these species-specific secreted proteins contribute to the pathogenicity of *A. solani* HWC-168. However, we speculate that some of the proteins might function inside plant cells, which has been widely reported in the effector proteins of rust and oomycete plant pathogens [45–48]. Translocation of rust and oomycete effector proteins into plant cell largely depends on the conserved RxLx-motif [46, 47]. In the secretome of *A. solani* HWC-168, 12 RxLx-motif containing effector candidate proteins were found, which indicates that they might serve as virulence factors during *A. solani* infection. Recent reports showed that RxLR effectors from various fungal pathogens are involved in virulence, which will broaden the implications of our findings [49]. The RxLx-motif containing effectors involving in the pathogenicity during the interaction between *A. solani* HWC-168 and potato will be further investigated.

## Conclusions

In this study we developed and annotated the complete genome sequence of *A. solani* HWC-168, and predicted the conidiation-related genes and the secretome that contains the virulence-related genes. To our best knowledge, this is the first time that the effector candidate genes and conidiation-related genes have been predicted in the genome of *A. solani*, which will facilitate the identification and functional analysis of conidiation- and virulence-related genes in *A. solani*. Availability of the genome sequence of *A. solani* HWC-168 and its host potato coupled with advanced genetic and molecular approaches will enable an understanding of the molecular mechanisms underlying the *A. solani*-potato pathosystem.

## Methods

### Strain and culture conditions

The strain *A. solani* HWC-168 was isolated from the infected leaves of potato in Weichang County Hebei province in China. All *Alternaria* isolates were cultured on potato dextrose agar (PDA). Mycelia were obtained by growing the isolate for at least 7 days on PDA plates.

### Genomic DNA preparation and library construction

The mycelia were harvested by filtration and frozen at − 20 °C. DNA was extracted from the mycelia according to a modified etyltrimethylammonium bromide procedure (CTAB) [50]. Following DNA fragmentation, we constructed two genomic sequencing libraries: one is a paired-end library with 500 bp inserts and another is a mate-pair library with 5 kp insertion fragments. The paired-end library was constructed using the Paired-End DNA sample Prep Kit (Illumina, USA) following the protocols provided by the manufacturer. The mate-pair library was constructed using the Nexera Mate Pair Library Prep Kit (Illumina, USA) following the protocols provided by the manufacturer.

### Genome sequencing and assembly

Two constructed libraries were sequenced by using the Illumina GA II technology (Illumina, USA) Hiseq 2000 platforms at the Beijing Genomics Institute using the WGS (whole genome sequencing) method. The read length is 150 bp. Low-quality data containing a quality value of less than 20 and short reads (length 35 bp) were filtered from raw data by Dynamic Trim and Length Sort Perl program in the SolexaQA software [51]. SOAPdenovo software (http://soap.genomics.org.cn) was used to assembly sequences and gaps were immerged by SOAP Gap Closer software (http://soap.genomics.org.cn) [52]. ORFs (open reading frames) were predicted by Augustus 2.5.5 software [53], and were aligned with homologous proteins in the NCBI database (http://www.ncbi.nlm.nih.gov/). All confirmed ORFs were aligned with COG (Clusters of Orthologous Groups) in the NCBI database, and were classified by function based on alignment results and classification standards of COG. Repetitive elements were identified by CENSOR (http://www.girinst.org/censor) following the default parameters.

### Genome comparison

The genome comparison was performed among *A. solani* HWC-168, *A. arborescens* and *A. brassicicola*. Multiple sequence alignments of genomes were performed with Mugsy [54]. The homologous genes were aligned using *PanOCT* software [55] by designating the parameter values: protein sequence with > 60% identity, aligned length > 30% and E value less than 1e-5, the Intergenic distance was calculated using the method described previously [56].

### Secreted protein annotation and prediction

The secreted proteins putatively encoded in the genome of *A. solani* HWC-168 were predicted by SignalPv4.0 (http://www.cbs.dtu.dk/services/SignalP/), TMHMM-2.0 (http://www.cbs.dtu.dk/services/TMHMM-2.0/), TagetPV1.01 (http://www.cbs.dtu.dk/services/TargetP/), and Big-pi (http://mendel.imp.ac.at/gpi/fungi_server.html). In detail, we followed the effector prediction pipeline described previously [57]. We first searched the ones with the presence of an N-terminal signal peptide through signalIP4.1. Then, we excluded the ones with a predicted transmembrane domains using TMHMM-2.0. Next, we detected the presence of subcellular localization signals using TargetP and glycosylphophatidylinositol (GPI) anchor to the membrane and filtered out the ones with mitochondrial localization and then the ones with GPI. The secreted proteins in *A. arborescens* and *A. brassicicola* were predicted by using the same method*.* The secreted proteins in *A. solani* HWC-168 were used as inquiring sequences to search against the secretomes of *A. arborescens* and *A. brassicicola* by BLASTP. The parameter values were designated as E-value < 10^− 5^ and identity > 30%. The secreted proteins with no homologous ones in *A. arborescens* and *A. brassicicola* were defined as species-specific proteins in *A. solani* HWC-168*.*

### Carbohydrate-active enzyme annotation

All putative proteins of *A. solani* HWC-168 were searched against entries in the CAZy database by using CAZymes Analysis Toolkit [58] using the Carbohydrate Active Enzymes (CAZy) database (http://www.cazy.org). The parameter values were in default on the website. All identified proteins were then manually retrieved.

### Prediction of proteins with a RxLx[EDQ]

The predicted secreted proteins in *A. solani* HWC-168 were examined with the presence of the conserved host-targeting motif RxLx[EDQ]by using the MEME prediction server (http://meme-suite.org/tools/meme) with default parameters. The amino acids in the conserved RxLx[EDQ] motif were aligned by WEBLOGO software [59].

## Additional files

Additional file 1: Venn-diagram showing the cluster of orthologous group (COGs) genes for related three strains including *A. solani*, *A. arborescens* and *A. brassicicola.* Ortholog clusters were computed by using PanOCT with set parameter cutoffs (E value < 10^− 5^; match length > 30%; identity > 60%). (DOCX 137 kb)

Additional file 2: Representative enzymes with the same function but involving in different biological activities. (DOCX 14 kb)

Additional file 3: Twenty seven species-specific secreted proteins based on the prediction. (DOCX 17 kb)

Additional file 4: Three pairs of specific neighbor genes reside on three different scaffolds. (DOCX 14 kb)

Additional file 5: Conidia and conidiophores formed by *A. solani* HWC-168 were visualized under microscopy. (DOCX 154 kb)

## Abbreviations

CAZy: Carbohydrate-active enzymes
COG: Clusters of orthologous groups
CPP: Cerato-platanin protein
CTAB: Etyltrimethylammonium bromide
CWDEs: Cell wall degrading enzymes;
GPI: Glycosylphophatidylinositol
