# Supplementary material for: Genome sequence of the potato pathogenic fungus Alternaria solani HWC-168 reveals clues for its conidiation and virulence
Source: BMC Microbiol. 2018 Nov 6;18:176. doi: 10.1186/s12866-018-1324-3 (PMC6219093; doi:10.1186/s12866-018-1324-3)
Supplement: Supplementary file 1 — Venn-diagram showing the cluster of orthologous group (COGs) genes for related three strains including A. solani, A. arborescens and A. brassicicola. Ortholog clusters were computed by using PanOCT with set parameter cutoffs (E value < 10− 5; match length > 30%; identity > 60%). (DOCX 137 kb) [file 12866_2018_1324_MOESM1_ESM.docx]

**
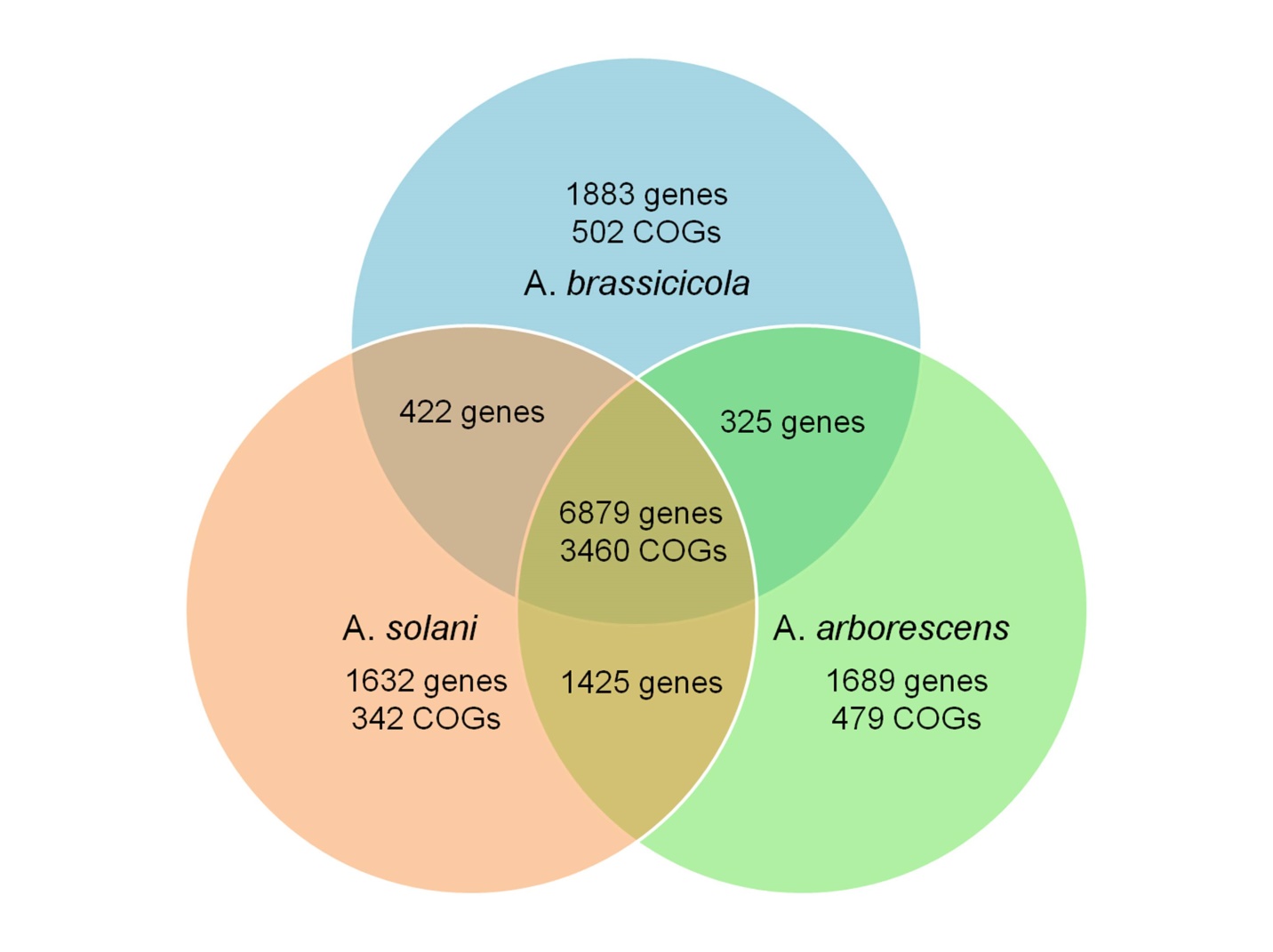
**

**Additional File 1.** Venn-diagram showing the cluster of orthologous group (COGs) genes for related three strains including *A. solani*, *A. arborescens* and *A. brassicicola.* Ortholog clusters were computed by using PanOCT with set parameter cutoffs (E value <10^-5^; match length >30%; identity >60%).
