# Supplementary material for: Genome sequence of the potato pathogenic fungus Alternaria solani HWC-168 reveals clues for its conidiation and virulence
Source: BMC Microbiol. 2018 Nov 6;18:176. doi: 10.1186/s12866-018-1324-3 (PMC6219093; doi:10.1186/s12866-018-1324-3)
Supplement: Supplementary file 2 — Representative enzymes with the same function but involving in different biological activities. (DOCX 14 kb) [file 12866_2018_1324_MOESM2_ESM.docx]

**Additional File 2.**

**Table S1 Representative enzymes with the same function but involving in different biological activities**

| Description | Physiological activity |
| --- | --- |
| Probable endo-beta-1,4-glucanase D | Developmental process |
|  | Mycelium development |
|  | Anatomical structure development |
| Polygalacturonase | Starch and sucrose metabolism |
| Pectate lyase  Probable glucan endo-1,3-beta-glucosidase eglC  Agglutinin isolectin | Pentose and glucuronate interconversions  Metabolic pathways  Biosynthesis of secondary metabolites  Ribosome  RNA degradation  Nucleotide excision repair  Metabolic pathways  Oxidative phosphorylation  Nucleotide excision repair  Endocytosis  Glycerophospholipid metabolism  Biosynthesis of secondary metabolites  Phagosome  Steroid biosynthesis |
