# Supplementary material for: Genome sequence of the potato pathogenic fungus Alternaria solani HWC-168 reveals clues for its conidiation and virulence
Source: BMC Microbiol. 2018 Nov 6;18:176. doi: 10.1186/s12866-018-1324-3 (PMC6219093; doi:10.1186/s12866-018-1324-3)
Supplement: Supplementary file 3 — Twenty seven species-specific secreted proteins based on the prediction. (DOCX 17 kb) [file 12866_2018_1324_MOESM3_ESM.docx]

**Additional File 3.**

**Table S2. 27 species-specific secreted proteins based on prediction.**

| **Name** | **Size** | **Scaffold** | **Scaffold Size (bp)** | **Chromosome number of *A. solani* altNL03003** |  |
| --- | --- | --- | --- | --- | --- |
| ASLO__50 | 95 | 4 | 167609 | 1 |  |
| ASLO__71 | 281 | 4 | 167609 | 1 |  |
| ASLO__189 | 133 | 10 | 232400 | 3 |  |
| ASLO__764 | 733 | 18 | 1010208 | 1 |  |
| ASLO__791 | 123 | 18 | 1010208 | 1 |  |
| ASLO__1448 | 189 | 13 | 1607436 | 10 |  |
| ASLO__2075 | 122 | 29 | 1649472 | 7 |  |
| ASLO__2487 | 121 | 53 | 1722612 | 9 |  |
| ASLO__2599 | 252 | 53 | 1722612 | 9 |  |
| ASLO__2683 | 181 | 24 | 2495362 | 8 |  |
| ASLO__2994 | 463 | 24 | 2495362 | 8 |  |
| ASLO__3140 | 323 | 24 | 2495362 | 8 |  |
| ASLO__3568 | 79 | 5 | 2567620 | 6 |  |
| ASLO__3685 | 202 | 5 | 2567620 | 6 |  |
| ASLO__4308 | 276 | 21 | 2613338 | 4 |  |
| ASLO__4866 | 229 | 21 | 2613338 | 4 |  |
| ASLO__4896 | 167 | 21 | 2613338 | 4 |  |
| ASLO__5162 | 69 | 1 | 2759359 | 5 |  |
| ASLO__5382 | 113 | 1 | 2759359 | 5 |  |
| ASLO__7160 | 250 | 8 | 4985952 | 2 |  |
| ASLO__7214 | 66 | 8 | 4985952 | 2 |  |
| ASLO__7218 | 135 | 8 | 4985952 | 2 |  |
| ASLO__7586 | 143 | 8 | 4985952 | 2 |  |
| ASLO__8390 | 102 | 8 | 4985952 | 2 |  |
| ASLO__8543 | 99 | 8 | 4985952 | 2 |  |
| ASLO__8552 | 77 | 8 | 4985952 | 2 |  |
| ASLO__9621 | 278 | 15 | 5423972 | 1 |  |
