# Supplementary material for: Genome sequence of the potato pathogenic fungus Alternaria solani HWC-168 reveals clues for its conidiation and virulence
Source: BMC Microbiol. 2018 Nov 6;18:176. doi: 10.1186/s12866-018-1324-3 (PMC6219093; doi:10.1186/s12866-018-1324-3)
Supplement: Supplementary file 4 — Three pairs of specific neighbor genes reside on three different scaffolds. (DOCX 14 kb) [file 12866_2018_1324_MOESM4_ESM.docx]

**Additional File 4.**

**Table S3. Three pairs of specific neighbor genes reside on three different scaffolds.**

| Group | Scaffold N. Scaffold Size (bp) | Protein |
| --- | --- | --- |
| 1 | scaffold 18 1010208 | ALSO_764 |
|  |  | ALSO_791 |
| 2 | scaffold 21 2613338 | ALSO_4866 |
|  |  | ALSO_4896 |
| 3 | scaffold 8 4985952 | ALSO_7214 |
|  |  | ALSO_7218 |
