# Supplementary material for: Genome sequence of the potato pathogenic fungus Alternaria solani HWC-168 reveals clues for its conidiation and virulence
Source: BMC Microbiol. 2018 Nov 6;18:176. doi: 10.1186/s12866-018-1324-3 (PMC6219093; doi:10.1186/s12866-018-1324-3)
Supplement: Supplementary file 5 — Conidia and conidiophores formed by A. solani HWC-168 were visualized under microscopy. (DOCX 154 kb) [file 12866_2018_1324_MOESM5_ESM.docx]

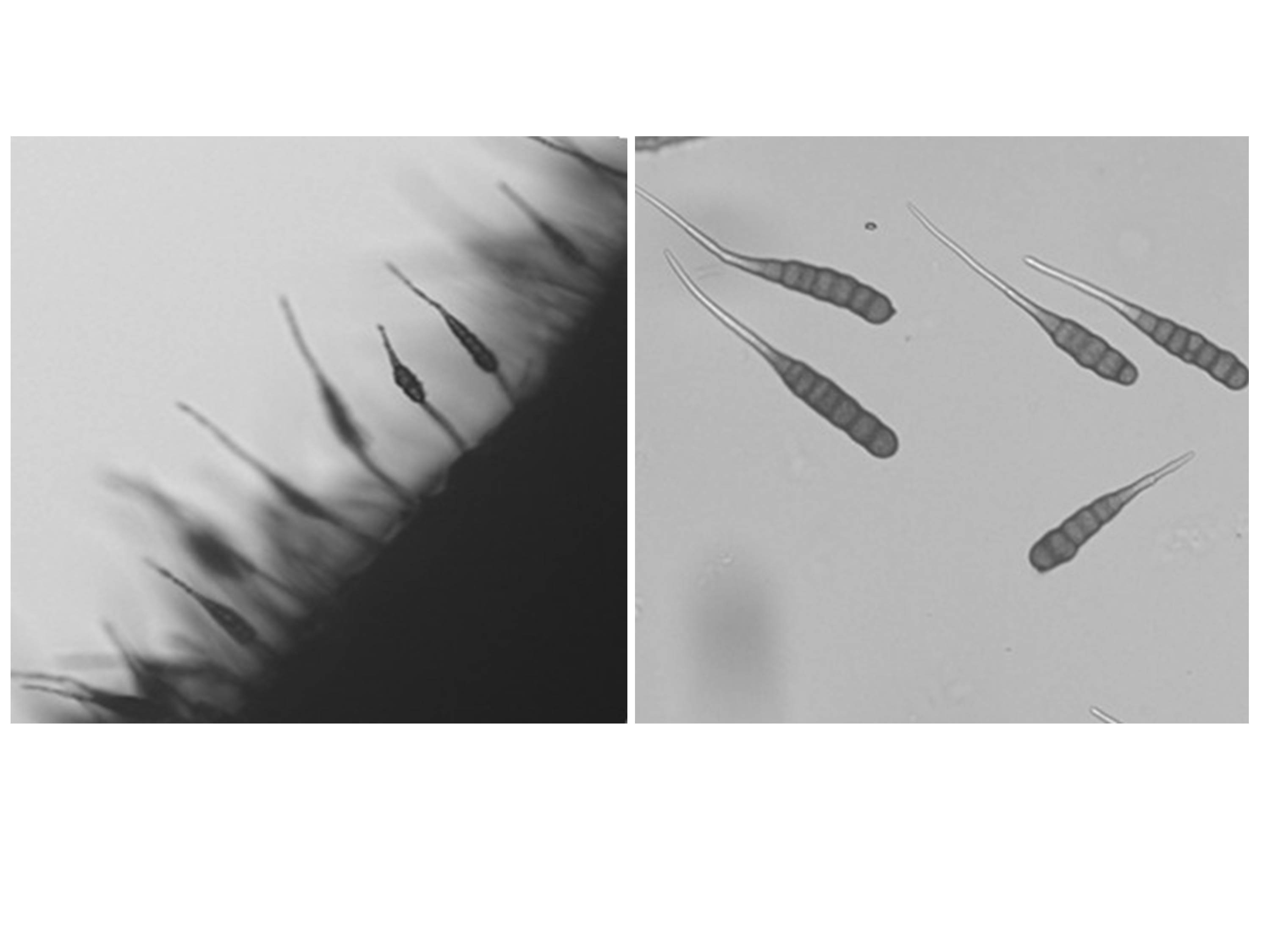
**Additional File 5. Conidia and conidiophores formed by *A. solani* HWC-168 were visualized under microscopy.**
